# Functional Traits of *Pinus ponderosa* Coarse Roots in Response to Slope Conditions

**DOI:** 10.3389/fpls.2019.00947

**Published:** 2019-07-30

**Authors:** R. Kasten Dumroese, Mattia Terzaghi, Donato Chiatante, Gabriella S. Scippa, Bruno Lasserre, Antonio Montagnoli

**Affiliations:** ^1^Rocky Mountain Research Station, Forest Service, U.S. Department of Agriculture, Moscow, ID, United States; ^2^Department of Biotechnology and Life Science, University of Insubria, Varese, Italy; ^3^Department of Biosciences and Territory, University of Molise, Pesche, Italy

**Keywords:** functional root traits, I-beam, root cage, root system architecture, root topology, T-beam, tree anchorage

## Abstract

We excavated the root systems of *Pinus ponderosa* trees growing on a steeply sloped, volcanic ash-influenced soil in the northern Rocky Mountains of the United States to assess their functional coarse-root traits and root system architecture. Trees, outplanted as one-year-old seedlings from a container nursery, were in their 32nd growing season on the site. We found that the trees had deployed more roots, in terms of length and volume, in the downslope and windward quadrants than in their upslope and leeward quadrants, likely a response to mechanical forces toward improving stability. Moreover, we observed the development of three types of root cages (tight, enlarged, and diffused) that likely reflect micro-site characteristics. As the cage type transitioned from tight to enlarged to diffused we measured a decrease in the overall volume of the roots associated with the cage and the taproot becoming a more prominent contributor to the overall volume of the cage. Finally, we noted the development of specialty roots, namely those with I-beam and T-beam shapes in cross section, in the downslope quadrant; these types of roots are known to better counteract compression mechanical forces. These observations improve our understanding of root plasticity and tree rooting response to environmental stimuli, which is becoming an increasingly critical topic as changes in climate increase the frequency and intensity of storms.

## Introduction

The global need for forest restoration continues to increase; attempting to meet that challenge is a host of current initiatives spanning scales from local to global and addressing nearly 500 million hectares (see Haase and Davis, 2017). This focus is not surprising given that terrestrial forest ecosystems cover about one-third of the global land base and account for 70% of the carbon exchange (Waring and Schlesinger, 1985) that occurs in the biosphere. Moreover, forest ecosystems support biodiversity (Pawson et al., 2013), which is important for maintaining ecosystem resilience to changes in climate (Liang et al., 2016; Seidl et al., 2016), and sustaining social structure (Parrotta et al., 2012).

To ensure long-term results, forest restoration activities must consider current conditions as well as future, uncertain climatic conditions (e.g., Millar et al., 2007). For example, extreme weather events (i.e., drought and windstorms) that occur with greater year-to-year variation are expected to occur more frequently or with greater severity (Meehl et al., 2000; Harris et al., 2006; Allen, 2009; Allen et al., 2010). This short-term change to climate, along with longer-term changes to climatic means (i.e., temperature and precipitation) are likely important drivers of forest degradation (Stanturf et al., 2014) that will increase the need for restoration across all scales (Chen et al., 2011).

Despite a strong effort during the last few decades to better understand the contribution of the belowground portion of trees to the biosphere response to global change, advancement of our knowledge concerning roots moves slowly due to the inherent difficulty in measuring these complex structures (Nadelhoffer and Raich, 1992). Critical gaps in our knowledge of root traits remain. An exact estimation of all traits belonging to a root system is, however, necessary for correctly modeling distribution of the mechanical forces involved in tree anchorage to soil.

A complete knowledge of all the anchorage properties of trees could enable us to predict the response of trees to more severe, climate-change induced storms, as well as to inform silvicultural practices, such as thinning, toward improving the resilience of existing forest stands facing increased drought events (Fraser and Gardiner, 1967; Ruel, 1995; Danjon et al., 1999a; Puhe, 2003; Crotteau and Ritchie, 2014), especially as new models are generated (e.g., Yang et al., 2016). The effects of thinning, in particular, are important because the increased distance between trees not only affects root development (Danjon et al., 1999b) but also changes the value of the mechanical loading because both slope and wind act on tree anchorage (Quine and Gardiner, 2007; Hale et al., 2012; James et al., 2014).

Individual roots, as well as root location, promote the effective anchorage of trees. Strong anchorage near the tree base utilizes four different cross-sectional shapes of large roots: circular, oval, I-beam, and T-beam (Nicoll and Ray, 1996; Coutts et al., 1999). In particular, I- and T-beam are identified as nonsymmetrical, secondary thickening around the vertical axis through the biological center. For an I-beam, equal vertical thickening occurs above and below the biological center. The T-beam shape is characterized by an uneven lateral thickening between the upper and lower regions of the root. On sites with shallow soil and in young trees, T-beam shaped roots tend to develop close to the stem base on the leeward side. I-beam shaped roots tend to develop on the windward side approximately 2.5X farther out from the stem base than the T-beam shaped root area on the leeward side (Nicoll and Ray, 1996; Coutts et al., 1999). Both of these root shapes move the focal point of bending/hinging farther away from the stem (Nicoll and Ray, 1996; Stokes, 1999; Chiatante et al., 2003). Development of an I-beam root shape increases root stiffness nearly 300X compared to a circular-shaped root having an equal cross-sectional area (Nicoll et al., 2006b). Trees, to maintain anchorage by resisting vertical flexing, tend to develop oval or I-beam roots in response to steep slopes and wind (Nicoll and Ray, 1996; Coutts et al., 1999; Chiatante et al., 2003; Di Iorio et al., 2005). I-Beam and T-beam shaped roots were found on *Betula* spp., *Picea sitchensis*, *Pinus contorta*, *Pinus sylvestris*, and *Quercus* spp. (Anonymous, 1964; Nicoll and Ray, 1996).

The arrangement of roots into a “cage” also affects anchorage (Danjon et al., 2005). The cage is defined as a cylindrical region centered at the taproot and composed of the zone of rapid taper of horizontal surface roots as well as the numerous sinkers and deep roots that enmesh a large mass of soil. The formation of a rigid cage is common in mature *Pinus pinaster* trees (Danjon et al., 2005). As is the case with trees that develop a rigid root-soil plate through adaptive growth of their structural roots to increase the contribution of soil resistance to overturning (Coutts, 1986; Ray and Nicoll, 1998), *P. pinaster* with low cage volume are more susceptible to wind-throw than their cohorts having a larger volume of leeward roots within the cage (Danjon et al., 2005).

*Pinus ponderosa* is one of the most important commercial species in the United States, covering about 10.9 million ha in the west (Oliver and Ryker, 1990). While a large body of literature has been accumulated for this species, most studies have focused mainly on above-ground characteristics (e.g., Weaver, 1961; Agee, 1993; Covington and Moore, 1994; Fulé et al., 1997; Moore et al., 2004; Wright and Agee, 2004; Hessburg et al., 2005), leaving below-ground structures much less studied, especially for trees beyond the seedling stage. Notable exceptions are the works published and referenced in Curtis (1964), and a large-root biomass model based on stem diameter at breast height (DBH; ~1.3 m) (Omdal et al., 2001). At the seedling stage, the ability of *P. ponderosa* to establish better than other conifers during periods of soil moisture deficits has long been linked to the adaptability of their root systems (e.g., Larson, 1963, 1967; Larson and Schubert, 1969; Smith, 1985; Kolb and Robberecht, 1996), but, to the best of our knowledge, no empirical work has been done to understand the root system architecture of more mature trees. A better understanding of the development and deployment of root system in its rooting environment could have important implications in the effort to improve the resilience of these forests and to preserve them within a scenario of changing global climate.

We hypothesized that coarse roots would have asymmetric spatial distribution influenced by the main mechanical forces of slope and prevailing wind. A second hypothesis was that the main coarse roots of *P. ponderosa* would display an I- and/or T-beam shape in response to these forces. To test our hypothesis, length and volume of the coarse roots were analyzed as a function of their spatial distribution into soil, and main shallow roots were sectioned proximal to the taproot and their cross-sectional shapes observed. Our objective was to use an information-theoretic approach to understand how *P. ponderosa* trees modify the growth of their root systems in slope conditions on an ash-cap soil type to adjust to different rooting environment stimuli.

## Materials and Methods

### Site Description and Tree Establishment

The study site is located at about 1,000 m elevation on the University of Idaho Experimental Forest in northern Idaho USA (lat 46.842240, long −116.871035). The area receives about 965 ml of annual precipitation with a seasonal drought during summer (July–September). The average, annual air temperature is 7.2°C with ~100 frost-free days (Soil Survey Staff, 2013). The prevailing wind during the growing season is west southwest (Western Regional Climate Center, 2019). Ecologically, the site is classified as a *Thuja plicata*/*Clintonia uniflora*/*Clintonia uniflora* phase habitat type (Cooper et al., 1987) that supports mixed conifer forests. It has a northeast aspect with slopes of 30–50%. The deep (~1.5 m) soil is in the Vassar series (Typic Udivitrands; Andisol), having formed in volcanic ash above material weathered from granitic (Soil Survey Staff, 2013); see Table 1 for profile descriptions. The site was clearcut harvested and broadcast burned during 1985 with little reduction of the forest floor layer.

**Table 1:** Profile characteristics of a typical Vassar series profile (National Cooperative Soil Survey, 2019).

Horizon	Descriptions of horizon abbreviations	Depth (cm)	Texture	Bulk density (g cm^3^)	Rock content > 2 mm (%)	pH	Carbon (%)	Nitrogen (%)
Oi	(O) Organic layer (i) Slightly decomposed organic matter	0–3	–	–	5	6.8	47.86	1.61
A	(A) Mineral; organic matter (humus) accumulation	3–10	Ashy silt	0.94	5	6.3	3.46	0.19
Bw1	(B) Subsurface accumulation Fe, Al, Si (w) Weak color or structure (1; suffix) horizon subdivision	10–24	Ashy silt	0.97	6	6.2	1.61	0.09
Bw2	(2; suffix) horizon subdivision	24–60	Ashy silt	0.99	4	6.3	1.20	0.07
2Bw3	(2; prefix) lithologic discontinuity (B) (w) (3; suffix) horizon subdivision	60–77	Silt	1.53	9	6.3	0.11	0.02
2BC	(2; prefix) (BC) Dominantly B characteristics but contains C horizon attributes	77–102	Loamy sand	1.56	14	6.1	0.07	0.01
2C	(2; prefix) (C) Little or no pedogenic alteration	102–136	Loamy sand	1.53	11	6.0	0.06	0.02
2Cr	(2; prefix) (C) (r) Weathered or soft bedrock	136–150	Coarse sand	–	5	6.1	0.04	0.01
Bedrock	Granite							

During March 1986, one-year-old *P. ponderosa* seedlings grown at the University of Idaho nursery in two container types (using locally collected seeds) were hand-planted on the site as part of an experiment (see Wenny et al., 1988). Twenty non-treated control seedlings of each container type were part of this outplanting in a grid having 1-m spacing between seedlings within the row (a single treatment/container combination) and 2-m spacing between rows. Each seedling was marked with a metal stake. During September 1986, every other seedling was excavated to observe first-season shoot and root growth (see Wenny et al., 1988), leaving residual trees on a 2 m × 2 m spacing. During this sample, the average bulk density, organic matter content, and pH in the rooting zone, defined as the top 25 cm of mineral soil, were determined to be 0.94 g cm^−1^, 4.7%, and 5.9, respectively (Wenny et al., 1988). No irrigation, fertilization, weeding, or thinning was done after outplanting.

### Excavation and 3-Dimensional Architecture Measurement

In early July 2017, we relocated the *P. ponderosa* trees grown in the control Styroblock 4A (313A) containers (60 ml volume, 14 cm depth, 936 cavities m^−2^; Beaver Plastics Ltd., Acheson, AB, Canada) and randomly selected five trees for measurement (P1, P4, P5, P6, and P8). Each tree was measured for DBH (cross-slope). Using the sample tree as plot center, we measured the azimuth, distance to, and DBH of other trees (>5 cm DBH) within a 5 m radius. A single screw was driven into the bark at the root-stem interface to delineate north. After cutting the stem near the collar, we measured height. Two screws were partially drilled into the stump about 20 cm apart with their heads adjusted to horizontal level. Two more screws, perpendicular to the first two, were installed in a similar manner.

We excavated the root systems using a high-pressure air lance fitted with a 71 l s^−1^ nozzle (AirSpade 2000; AirSpade, Chicopee, MA, USA) connected to a portable air compressor (36.5 kW) that delivered air at 87 l s^−1^ at a normal effective working pressure of 0.7 MPa (XAS 185; Atlas Copco Compressors LTD, Rock Hill, SC, USA). When the resulting supersonic air stream touched a smooth object (such as a stone or root), it slipped over the surface, but when the air stream hit any tiny pore, air was compressed in the pore (it could not blow out under such a high air speed) and the pore exploded. Thus, soil was blown away while the roots and other smooth objects remain untouched (Nadezhdina and Čermák, 2003). We exposed root systems to bedrock (approximately 1–1.5 m in depth) and to distances of approximately 1.5 m from the trunk. After cutting roots that were still attached to soil, the root systems were carefully lifted from the soil and carried to the U.S. Department of Agriculture, Rocky Mountain Research Station, Forestry Sciences Laboratory (Moscow, ID) for analysis. At the laboratory, we positioned the root systems on four adjustable wood supports so that the exact inclination (achieved by adjusting the root so that the screw heads were at horizontal level) and north direction (positive X; see below) could be restored.

The root system was discretized by a low magnetic field digitizer (Fastrak; Polhemus, Colchester, VT, USA) and encoded in a standard format (MTG) commonly used for representing branching topological relationships using AMAPmod software at different observation scales (Godin and Caraglio, 1998). Device characteristics (Danjon et al., 1999b; Di Iorio et al., 2013) consisted of an electronic unit, a magnetic transmitter (Long Ranger; Polhemus), and a small hand-held receiver positioned at each point to be measured. The receiver measured the X, Y, and Z spatial coordinates within a sphere-wide electromagnetic field having a 4-m radius around the transmitter, which was sufficient for the root system sizes observed in this study. The transmitter was positioned approximately 1.5 m below and 2.5 m from the stump with the downslope direction in the positive X direction.

**Figure 1:**
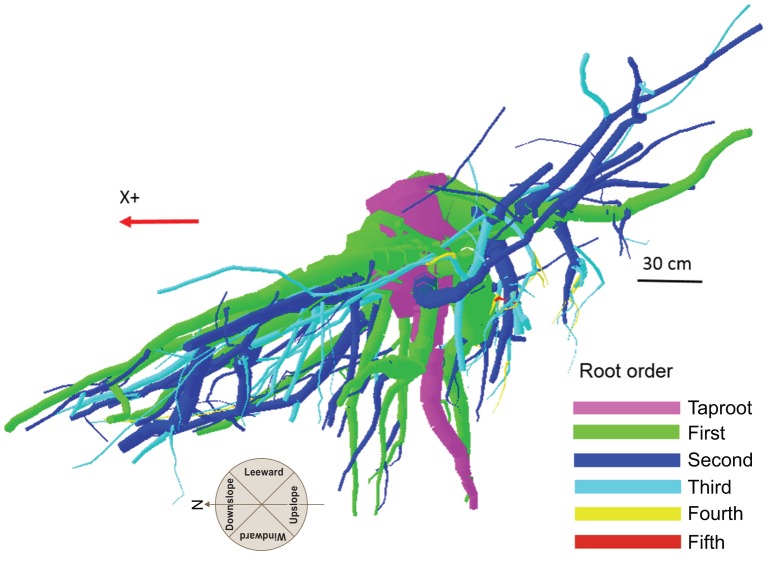
Root hierarchy was digitized using the “acropetal-development approach” that allows each root system to be reconstructed using the AMAPmod software for 3-dimensional analysis. In this image of tree P8, the taproot appears pink, first-order lateral roots emerging from the taproot are green, and second-order roots are blue. The X + axis is oriented down slope parallel to the slope direction.

Although scientists working with the finest component of the root system define the first-order roots as those most distal (McCormack et al., 2015), in the present work the topology, (i.e., the branching hierarchic structure) was coded according to the “acropetal-development approach” (Danjon et al., 2013; Sorgonà et al., 2018) with the seed-origin radicle, the primary roots (-axis) or taproot designated order zero (pink color in Figure 1). Lateral roots emerging from the taproot were designated first-order roots (green color in Figure 1), with second-order roots then originating from these first-order laterals (blue color in Figure 1), and so on (Zobel and Waisel, 2010). The stump was determined subjectively as the portion of taproot with a large diameter from where most of the large horizontal surface roots originated. The taproot was the largest vertical root originating directly from the stump. We digitized starting at the root collar and followed a recursive path along the branching network as suggested by Danjon et al. (2005). Between branching points, intermediate measurements were performed in order to record changes in root direction and taper. A segment was defined as the root subdivision between two measured points. The average segment length was about 2 cm when roots were curved and approximately 15 cm when roots were straight. When a root cross-section was oblong, the largest diameter and its orientation, as well as the diameter perpendicular to the largest diameter, were recorded. All roots with a proximal diameter larger than 1 cm at the base were measured.

**Figure 2:**
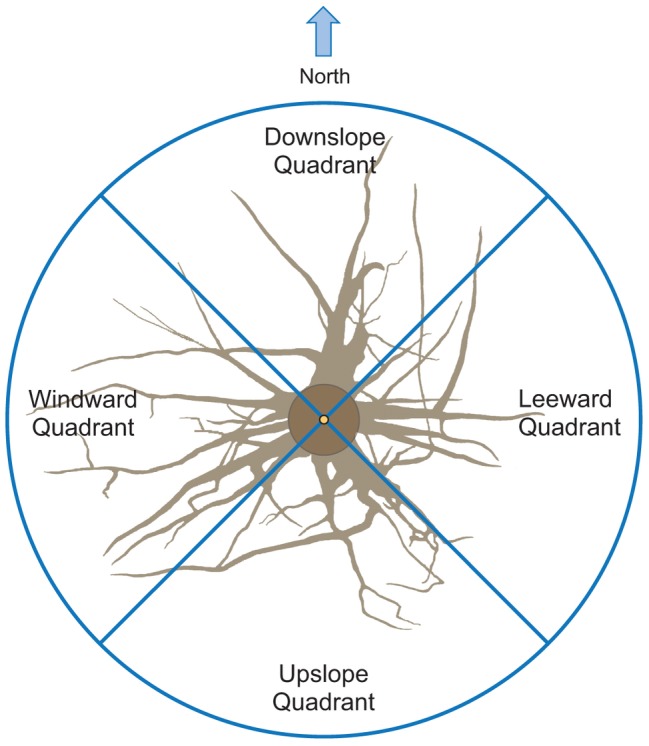
Roots were excavated to a distance of about 1.5 m from the trunk. Traits were assessed in four quadrants: upslope, downslope, windward, and leeward.

The output data file was analyzed using the AMAPmod software (Godin et al., 1997), which handles topological structure at several scales and also provides 3-dimensional graphical reconstruction for data checking. Extracted data were exported to other software to perform specialized processing (see statistical analysis below). Root traits (i.e., length, diameter, and volume) were computed from 3-dimensional digitizing data of whole root systems. Root traits were considered as a function of up- versus down-slope direction and of west- versus east-slope direction, with the downslope direction coinciding with north and the west-east axis coinciding with the direction of the prevailing wind (Figure 2). Furthermore, the different root traits were assessed as a function of depth and azimuth position. After several analyses, we chose two depths for assessment: 0–30 and 30–60 cm. Within each depth, we divided the space surrounding the taproot into four quadrants: downslope (north), upslope (south), windward (west), and leeward (east).

We analyzed the root cage, defined as the cylindrical region centered at the taproot. The cage radius corresponds to the zone of rapid taper, which is calculated as the mean length of the first shallow root segments that extend from the stem base with the most rapid taper. The depth of the cage corresponds to that of the taproot (Danjon et al., 2005), and first-and second-order vertical roots were counted as sinker roots. In our study, we used an alternative definition, developed from Danjon et al. (2005) to relate the zone of rapid taper to tree size. Specifically, the zone was defined as all roots originating within a radial distance of 2.2 × DBH.

### Cross-Sectional Shape of Structural Roots

On each tree in the sector with the greatest root spatial allocations, cross-sectional samples from the largest shallow roots were cut at approximately the originating branching point from the taproot. Before cutting, the top of each root was labeled. One face of each root cross section was sanded smooth to allow examination of the growth rings.

### Statistical Analysis

To evaluate the effects of abiotic factors on root apparatus architecture, we compared the length and volume of first-, second-, and third-order roots using the IBM SPSS Statistics software version 20.0 (IBM Corp.; Armonk, NY, USA). Each depth was analyzed independently. The distribution of each population was tested using the Shapiro-Wilk normality-test. Parametric comparison methods were adopted if the result was positive; otherwise, we used non-parametric comparison tests. We used tests for related (non-independent) data to analyze group means. In particular, when variables were Gaussian distributed, we employed the paired samples *t* test; otherwise, the Wilcoxon signed-rank test was performed when variables were non-Gaussian distributed. Box-plot visualizations were created using SigmaPlot (version 13.0; Systat Software, San Jose, CA, USA). Root system projections were generated using PlantGL, a Python-based geometric library for 3-dimensional plant modeling at different scales (Pradal et al., 2009). Azimuth projection and graphical reconstruction of the tree stand characteristics were produced by Excel and PowerPoint (Microsoft Office 2003 Microsoft Inc., Redmond, WA, USA) software, respectively.

## Results and Discussion

The variability observed among the root systems of different tree species, and within a particular species, is considered to be an adaptation response to the variability of the rooting environment (i.e., depth and nature of soil) (Henderson et al., 1983; Coutts et al., 1990). Differences between *P. ponderosa* and the species it forms mixed stands with, such as *Populus tremuloides*, *Pseudotsuga menziesii*, and *Pinus contorta*, have been noted (Berndt and Gibbons, 1958), and differences in tap root development of *P. ponderosa* occurred because of varying soil depths to bedrock (Berndt and Gibbons, 1958; Curtis, 1964).

**Figure 3:**
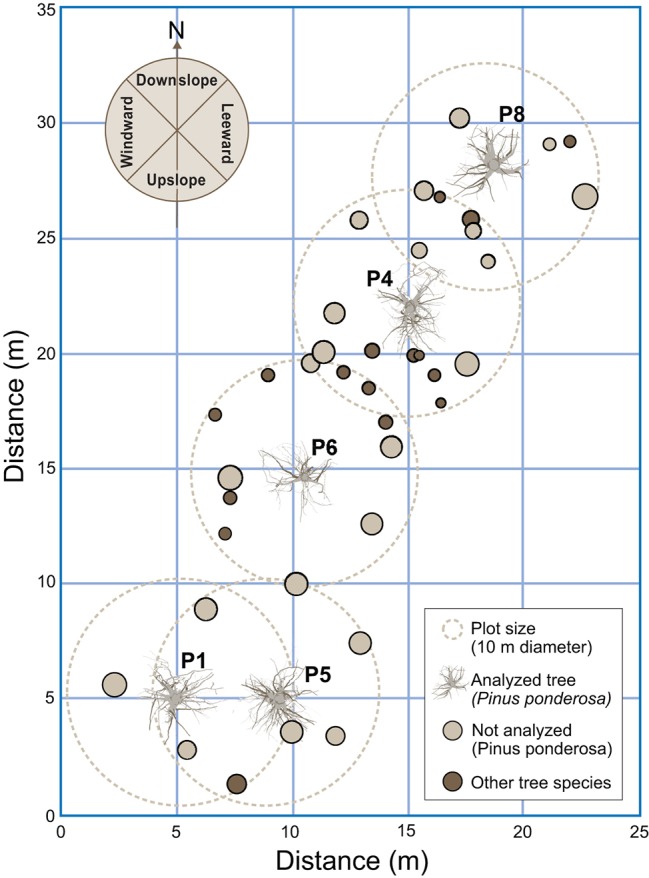
Locations of the sampled trees and their proximity to other trees. The other tree species included *Abies grandis*, *Larix occidentalis*, and *Pseudotsuga menziesii*. Downslope was to the north and windward was to the west.

In our study, however, the sampled area was small (132 m^2^) and the trees fairly close (~2 m) (Figure 3). Therefore, it is reasonable to assume that the soil was uniform in terms of slope, aspect, and profile across the sampled area. Thus, any differences observed in overall root traits and architecture may represent a response of the trees to differences existing in the soil profile relative to physical and chemical properties.

### Root System Traits

Overall, DBH of sampled trees ranged from 23.3 to 34.2 cm, with a median value of 24.1 cm (Table 2). Heights were about 16 m. Variability in root length increased with root order (topology follows the “acropetal-development approach”; Danjon et al., 2013; Sorgonà et al., 2018); that is, more variability was observed in second-order roots compared to first-order, and more in third-order roots compared to second-order (Table 2). While the trend was less apparent for root volume, the third-order roots were again the most variable.

**Table 2:** Above- and below-ground characteristics for each analyzed tree.

Tree	DBH (cm)	Height (m)	Length (cm)			Volume (cm^3^)		
			Root order					
			First	Second	Third	First	Second	Third
P1	24.1	16.8	2,994	4,255	1,033	74,550	10,771	703
P4	24.1	15.2	2,859	7,482	2,492	50,640	18,192	2,099
P5	25.6	16.0	2,429	8,536	5,314	57,596	25,648	7,104
P6	23.3	17.1	3,180	4,510	1,744	30,275	22,117	1,777
P8	34.2	16.2	3,310	5,028	1953	165,406	35,263	6,826
Mean	26.3	16.3	2,954	5,962	2,507	75,693	22,398	3,702
SE	2.0	0.3	152	860	738	23,483	4,049	1,350

*Total length and volume were for roots with a diameter > 1 cm*.

**Figure 4:**
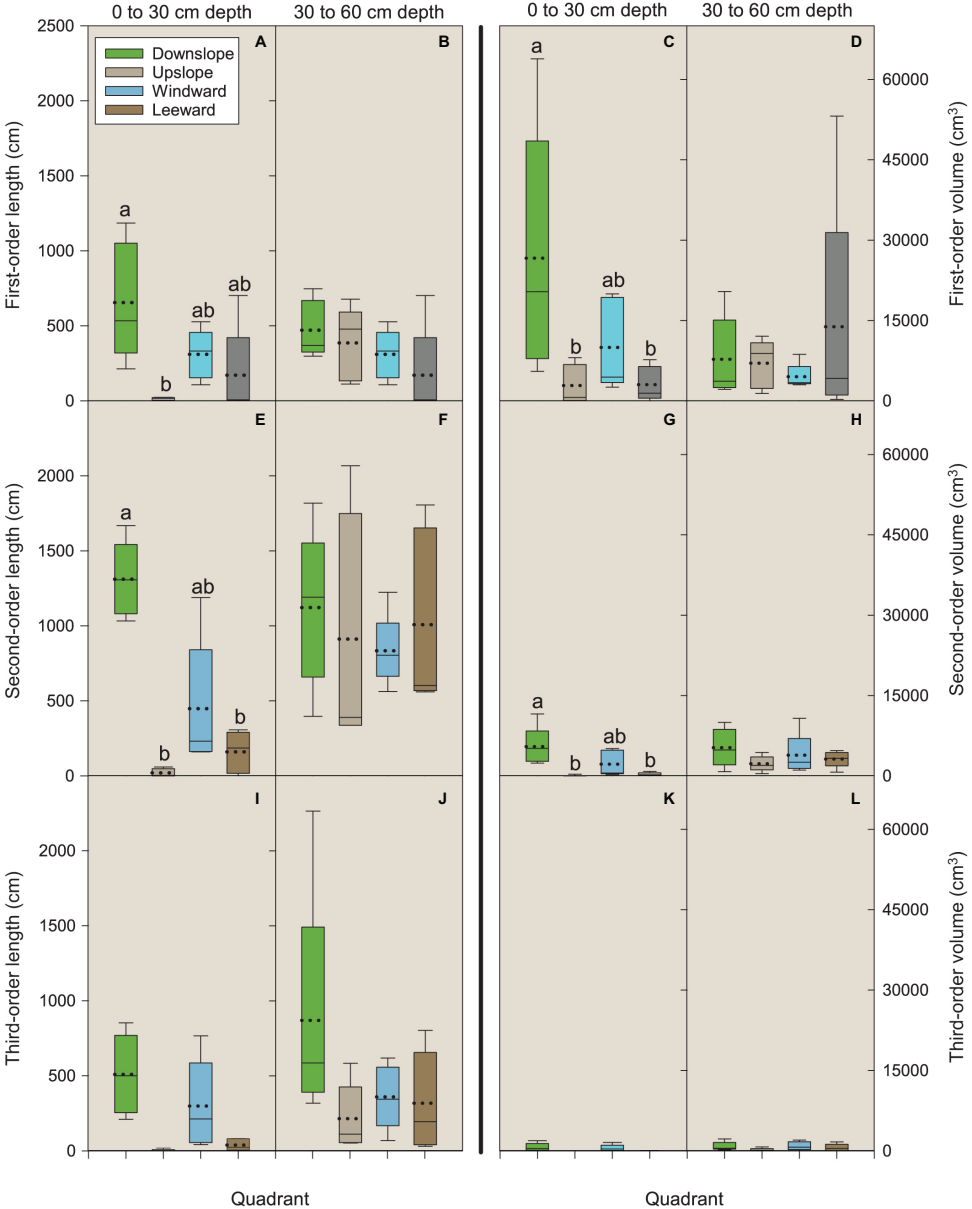
Length and volume of first-, second-, and third-order roots by quadrant and soil depth (0–30 and 30–60 cm). Each depth was analyzed independently. Letters within each order, variable, and depth combination indicate significant differences (*p* < 0.05); absence of letters reflects that no significant difference was detected. Vertical boxes represent approximately 50% of the observations and lines extending from each box are the upper and lower 25% of the distribution. Within each box, the solid horizontal line is the median value and the dotted line is the mean.

In the Vassar soil series, a lithological discontinuity occurs at about the 60 cm depth in a typical profile. At that depth, the influence of the volcanic ash dissipates, bulk density increases from about 1 to 1.5 g cm^−3^, and percentage carbon (a reflection of overall organic matter) drops from 1.2 to 0.1% (Table 1). Above this discontinuity, the soil depth of 0–30 cm includes more carbon (organic matter) and nitrogen than the lower, 30–60 cm depth (Table 1) and this upper zone is where significant differences in root length and volume were observed (Figures 4A,C,E,G).

For first-order lateral roots in the 0–30 cm depth, length in the downslope quadrant was significantly different (*p* = 0.0225) than that observed in the upslope quadrant (Figure 4A). For volume, the downslope quadrant was significantly different than the leeward (*p* = 0.0425) and upslope (*p* = 0.0490) quadrants (Figure 4C). No significant differences were noted for length or volume in the 30–60 cm depth (Figures 4B,D).

For second-order lateral roots in the 0–30 cm depth, length in the downslope quadrant was significantly different than the leeward (*p* = 0.0430) and upslope (*p* = 0.0126) quadrants (Figure 4E). Volume followed the same pattern as that observed for first-order roots: the downslope quadrant was significantly different than the leeward (*p* = 0.0422) and upslope (*p* = 0.0352) quadrants (Figure 4G). No significant differences were noted for length or volume in the 30–60 cm depth (Figures 4F,H). We were unable to detect any significant differences among third-order lateral roots for length or volume in the 0–30 cm depth (all *p* ≥ 0.6) (Figures 4I–L).

Trees grow roots in response to environmental stimuli (Rellán-Álvarez et al., 2016). On our study site, two possible displacement forces may be influencing root occurrence: prevailing wind and slope (Chiatante et al., 2003; Danjon et al., 2005; Di Iorio et al., 2005; Lombardi et al., 2017). Stokes (2002) notes that for trees undergoing mechanical stress due to the force of unidirectional wind, the roots perpendicular to the direction of possible displacement (windward-leeward direction) are held in torsion and play a marginal role in counteracting uprooting forces (Stokes, 2002). On our site with its 40% slope, it is, however, reasonable that the mechanical forces act with an upslope-downslope direction; thus more roots should grow in the upslope-downslope direction than in the windward-leeward direction. Indeed, our data indicate that two contemporaneous mechanical forces affect root spatial development: slope and prevailing wind. Lower values of first-and second-order root length and volume in the leeward and upslope quadrants suggest that the anchorage of *P. ponderosa* trees excavated in this study preponderantly rely on roots belonging to the downslope and windward quadrants. The latter being of lower magnitude because the windward root traits were not significantly different than the leeward side despite an observed trend. Thus, slope induces mechanical force acting on the roots with an upslope-downslope orientation (Chiatante et al., 2003; Scippa et al., 2006; Sun et al., 2008), while mechanical forces due to the wind act in the windward-leeward direction (Yang et al., 2014 and references therein). Furthermore, although the magnitude of root volume and length in the 30–60 cm soil depth is comparable to those in the 0–30 cm depth, at the deeper soil layer differences in quadrants were not detected. This finding supports our first hypothesis and indicates a probable interplay between mechanical forces, higher N concentrations, and lower bulk density that occurs in the upper part of the soil profile. These factors influence the displacement of surface roots that are important for dissipating the tree “self-loading” to the soil (Chiatante et al., 2003) because of the higher entangled soil area as well as, during the juvenile stage, for water and nutrient absorption.

Our results are opposite to those reported for an oak tree species (*Quercus pubescens*; Di Iorio et al., 2005) and a common Mediterranean shrub species (*Spartium junceum*; Lombardi et al., 2017) growing on clay soils in slope conditions; here the authors found less downslope biomass than upslope biomass. This adaptive growth behavior might be related to trees avoiding root growth into the drier portions of the soil profile. Indeed, on the downhill side on a steep slope, roots growing horizontally would grow into the drier upper soil profile and eventually emerge from the soil, but instead change direction due to decreasing soil moisture (Di Iorio et al., 2005). Thus, in this case and from a biomechanical point of view, the upslope roots’ resistance to pullout and shear stress might become the main component of tree anchorage. In other species, however, preferential growth of lateral roots occurred downslope (*Arabidopsis*, Mullen et al., 2005) or even perpendicular to the slope direction when a dominant wind was present (*Picea sitchensis*, Nicoll et al., 2006a; *Robinia pseudoacacia*, Khuder et al., 2006). In our study, the higher values of root traits were found downhill in accordance with different scenarios of root displacement outlined by Ghestem et al. (2011). Indeed, from a biomechanical point of view, preferential root growth occurs either up- or down-slope, thus enhancing anchorage along the axis of static mechanical loading (Stokes et al., 2009). In our case and on one hand, the anchorage of the tree is likely attributable to the forces of the roots pushing downward rather than hanging upward. On the other hand, certainly, these roots play an important role in the exploration and exploitation of surface water and nutrients during the juvenile developmental stage, thereby shaping future root spatial displacement. Thus, from a hydrological perspective, more roots are oriented downslope than upslope (Ghestem et al., 2011). This preferential gravitropism depends on the species, on nutrients, and on the soil’s physical properties.

Regarding the nature of these mechanical forces, we suggest that roots growing in the downslope and windward quadrants are subjected to compression forces, while roots in the upslope and leeward quadrants are subjected to tension forces. These forces are transferred to the soil *via* friction, so that a large root volume and length on both windward and downward side, over which the load can be distributed, is beneficial to tree anchorage (Stokes et al., 1996).

### Root System Architecture

It is known that root system architecture (RSA) exhibits a great variability due to genetic and environmental factors (see Gardiner et al., 2016 for a review; Schroth, 1998; Puhe, 2003; Stokes et al., 2009; Ji et al., 2012; Yang et al., 2016). On our site, *P. ponderosa* trees growing on an ashy silt soil show a highly structured RSA with coarse roots (diameter > 1 cm). In our study, trees displayed three RSA shapes (hereafter tight, enlarged, and diffused cage) where “cage” is intended to describe the zone of the root system around the stump where the taproot and most of the sinker roots descend into the soil in a parallel pattern (Danjon et al., 2005). These RSA shapes differ considerably from the heart-shape common to other conifers (Drexhage and Gruber, 1998; Stokes, 2002). However, the anomalous RSA shapes we observed concurs, as already discussed above, with the findings of Chiatante et al. (2003) and Di Iorio et al. (2005) for root systems developing on slopes.

**Figure 5:**
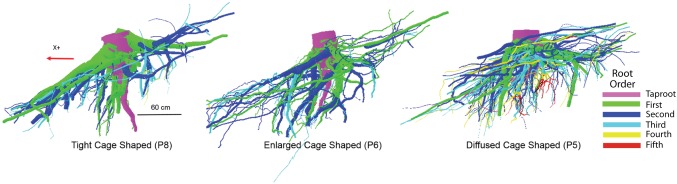
An example of the three different root system architectures (tight, enlarged, and diffused cages) related to differences in the zone around the stump where the taproot and most of the sinker roots descend into the soil in a parallel pattern. Different colors indicate differences in branching order.

In our study, the differences in RSA cage shapes are mainly dependent upon the variable proportion of number of sinkers, the volume of the first-, second-, and third-order roots within the cage, and the overall behavior of the taproot (Table 3). We observed three cage types: tight, enlarged, and diffused. A “tight cage” is characterized by a low number of sinker roots (P1 and P8 in Table 3) that form proximate the taproot (P8 in Figure 5). Furthermore, the ratio of the taproot to the volume of the entire cage is similar (0.39 and 0.37 respectively, Table 3), indicating a larger contribution of the sinker roots to cage volume, thus, to tree anchorage, in respect to the taproot. An “enlarged cage” is exemplified by a similar number of sinker roots (P6 in Table 3) that form proximate as well as distant from the taproot (P6 in Figure 5). Here the ratio of the taproot volume to the total cage volume is 50% showing an equal contribution by the taproot and the rest of the roots composing the cage to tree anchorage than that found in a tight cage. The highest number of sinkers (P4 and P5 in Table 3), located more distant from the taproot than other cage types, characterizes a “diffused cage” (P5 in Figure 5). For both P4 and P5 trees, the taproot volume is about 40% greater than the cumulative volume of the first-, second-, and third-order roots (Table 3; ratio of 0.59); here the taproot provides the greatest contribution to the cage and the overall anchorage of the tree compared to the other cage types.

**Table 3:** Characteristics of root system architecture for analyzed *Pinus ponderosa* trees: the number of first- and second-order sinker roots; the length and volume of the first-, second-, and third-order roots (>1 cm diameter) within a radius of 2.2 × DBH (diameter breast height); the length and volume of the taproot; the total cage volume (sum of first-, second-, and third-order roots and taproot); and the ratio of taproot volume to total cage.

Tree	Sinker roots (number)		First-, second-, and third-order roots		Taproot		Total	Taproot/total
	Order							
	First	Second	Length (cm)	Volume (cm^3^)	Length (cm)	Volume (cm^3^)	Volume (cm^3^)	Volume (cm^3^)
P1	6	13	2,254	63,786	116	41,169	104,955	0.39
P4	4	20	2,617	36,619	157	52,686	89,305	0.59
P5	2	22	3,406	49,913	272	72,258	122,171	0.59
P6	3	9	2,541	37,077	124	37,496	74,573	0.50
P8	6	11	3,762	144,529	165	86,195	230,724	0.37

Within a root system, the zone of rapid taper is a compartment including the portion of all the shallow roots that branch off from the taproot and undergo the most rapid decrease of diameter (Danjon et al., 2005). The zone of rapid taper along with the sinker roots plays a dominant role in tree anchorage with the taproot being the first mechanical contributor to tree anchorage strength (Yang et al., 2014, 2016). Nevertheless, we observed that, transitioning from the tight cage to the diffuse cage, total cage volume decreases while the contribution of the taproot to the root cage increases. This suggests that in *P. ponderosa*, the taproot and the rest of the cage roots contribute differently to tree anchorage depending on the high plasticity of the root at the individual plant level.

At an individual root level, we observed that shallow roots in the 0–30 cm soil depth in the downslope quadrant could develop both I- and T-beam shaped roots at their branching point from the taproot. This finding supports our second hypothesis. Compared to I-beam development, most roots (83%) in the down-slope quadrant displayed T-beam development. This adaptive growth strategy strengthens the anchorage because the T-beam is particularly well designed to resist compressive forces (Nicoll and Ray, 1996), which on our sites was likely caused by the force of gravity occurring downslope.

**Figure 6:**
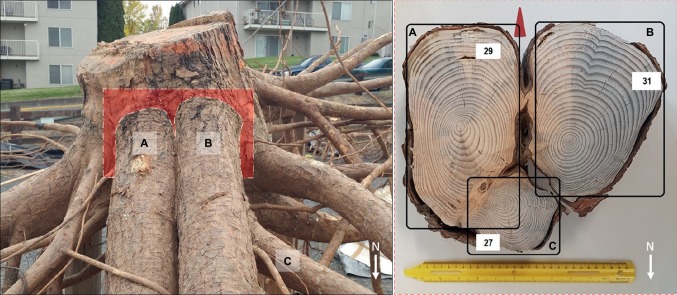
Tree P8 (left panel) with two first-order lateral roots **(A,B)** north-oriented (downslope) and a second-order root **(C)** bending eastward (leeward). These same three roots in cross section (right panel); both first-order roots are subjected to mechanical induction and show asymmetry in the rings with different shapes: I-beam **(A)** T-beam **(B)**. White arrows indicate the north direction and the red arrow in the right panel points toward the soil surface. The number of growth rings in each root is reported in the white boxes.

These cross sectional root shapes have been observed in conifer and broadleaved trees with shallow structural roots (Nicoll and Ray, 1996; Coutts et al., 1999; Nicoll et al., 2006a; Danjon et al., 2013) particularly in response to wind movement (Coutts et al., 1999). These shapes maximize resistance to bending or flexing and increase rigidity of the root-soil plate with minimum wood production (Coutts et al., 1999 and references therein). Therefore, in our case, this particular root shape could represent the response of trees to the need to increase their mechanical contribution to anchorage due to mechanical stimuli as a result of slope. We noted that roots with I- and T-beam shape were present in the downslope quadrant (Figure 6) along with the observation that root length and volume were also greatest in this quadrant. Thus, our findings concur with those of Stokes et al. (1996) that related the increase in buttress surface area of these roots obtained with either an I- or T-beam shape with a better counteracting of the compression mechanical forces by transferring those forces to the soil.

## Conclusions

Limited literature has discussed the root system architecture of *P. ponderosa*, with a paucity of information about this topic for trees growing on sloping ash-cap soils. On this site after 32 growing seasons, *P. ponderosa* trees appear to have deployed roots in response to mechanical forces due to both slope and prevailing wind by devoting more root resources downslope and windward toward improving stability. We noted growth of roots with I- and T-beam shapes in the downslope quadrant that better counteract the compression mechanical forces. Although this architectural pattern was common to all trees analyzed, we observed that the contribution of the taproot to the root cage, and thus to the tree anchorage, may vary depending on the plasticity of the root system in relation to the micro-soil conditions. Finally, these results unveil a powerful mechanism that involves modulation of root spatial displacement and morphology to increase tree stability. Thus, such an understanding of RSA provides useful information in terms of tree adaptation in the scenario of increasing frequency and intensity of storms.

## Data Availability

The datasets generated for this study are available on request to the corresponding author.

## Author Contributions

KD and DC conceived the research project. KD provided primary funding. AM, KD, and DC developed the study plan. AM was responsible for field excavations and data collection and analysis. AM and MT equally contributed to the field activities. AM performed the digitalization and provided the 3D visualization. BL performed the three-dimensional data arrangement and analysis. GS provided important insights into the study plan and research process. KD, DC, and AM prepared the manuscript.

### Conflict of Interest Statement

The authors declare that the research was conducted in the absence of any commercial or financial relationships that could be construed as a potential conflict of interest.
